# Account of a Peculiar Form of Malarial Pneumonia, with a Suggestive Plan of Treatment

**Published:** 1880-05

**Authors:** W. D. Hoyt

**Affiliations:** Rome, Georgia


### ACCOUNT OF A PECULIAR FORM OF MALARIAL PNEUMONIA, WITH A SUGGESTIVE PLAN OF TREATMENT.

By W. D. HOYT, M.D., Rome, Georgia.

We have been, and are still having in this place and vicinity, a form of malarial fever different from any I have before encountered, which has caused a number of deaths. These deaths have generally been attributed to the pneumonia which frequently accompanies the disease. A study of my cases, however, soon convinced me that the pneumonia was only an incident in the disease, as patients died just as soon, whether there was much or little of the lung involved, or no pneumonia at all. The first thing which attracted my attention was the obstinacy of the cases of pneumonia. The next was the peculiarity in the exacerbations. Instead of the usual exacerbations at noon and midnight, with remissions morning and evening, there was a single exacerbation occurring, either from the effect of treatment or from the nature of the disease at a later period each day. Another peculiarity was the height of the fever during an exacerbation—the temperature running up to 105, 105½ and 106, followed by a remission, when the temperature would fall to 103–4. The pulse was proportionally excited, running up to 120–40, and dropping during a remission to 102–10. Another peculiarity was the curious fact that the fatal cases, almost without exception, terminated on the seventh day of the disease. This was true whether there was double pneumonia, a half only of a lung involved, or in a case I left recently, simply bronchitis.

The onset of the disease is generally ushered in, in the case of adults, with a long-continued chill. With children this is not so marked. The chill is followed by the high fever; the tongue is inclined to dryness and is covered with a harsh brown coat. As the disease advances, pneumonia frequently develops, generally on the second or third day. About the fourth or fifth day typhoid symptoms manifest themselves, with subsultus tendinum cephalalgia musces volitantes and typhoid delirium, which is more pronounced during the exacerbation.

The malarial character of the disease was manifested by the periodical exacerbations, and yet quinine used most heroically failed to prevent these exacerbations. I was led to look into the cause of this ill-success with quinine, and recalled the case of a little boy who had a well-marked case of the disease, with pneumonia, in which case there was so much complaint of pain from an engorged liver, that I was led to apply a blister over that organ. The effect was most marked, and the recovery exceptionally rapid. I also recalled a conversation I had with that excellent physician and acute observer, Dr. C. H.

Gorman, of Alabama, who mentioned that he had treated cases of malarial fever in Alabama which would not yield to quinine until he blistered over the liver. I therefore determined to blister over the liver in every serious case, whether there was tenderness in that region or not, and to use calomel freely in all cases. Sincc arriving at this conclusion, I have treated eight well-marked cases of the disease, one adult and seven children. The adult, a woman with a chronic ailment which rendered her feeble, was attacked on Friday evening, April 23d, by the characteristic long-continued chill, followed by what was described as "the highest kind of fever."

I found her at 10 A.M., of Saturday, with a temperature of 103, (the fever having remitted) and the characteristic tongue. I directed a blister to be applied over the liver, gave her about fifteen grains of calomel, and directed her to take fifteen grains of quinine that evening and the same quantity the followiug morning. The next morning her husband reported that her bowels were being acted on every half hour, and that she was sick at the stomach and vomiting. I directed him to give her a pill of two-thirds of a grain of opium, which checked the vomiting and purging. At 11 A.M. I found the temperature 100. I directed mint julep and milk punch, with lime water, to be given and the quinine continued. On Monday she had no fever, and on Tuesday I dismissed the case, after directing her to continue the quinine in small doses, and to take some turpentine emülsions in consequence of the denuded condition of the tongue. To three of the cases among the children I had treated before this case, I now think, I did not give enough calomel. They all developed pneumonia, but have all recovered. One, I believe, owed his recovery to a repetition of the blister; to the other four I administered calomel more freely, and they at once recovered.

My belief as to the nature of these cases is, without an opportunity for post mortem examination, that the liver is intensely engorged, and that that prevents the quinine from producing its anti-periodic effect. This condition of engorgement has probably been produced by the exceptional character of the winter and spring. Several of the children complained of pains in the region of the liver, and some of pain in the shoulder. The object of the blister and the calomel is to disengorge the liver, when the fever will be readily controlled by quinine. The pain is sometimes more in the right, and sometimes in the left lobe of the liver, and the blister should be applied accordingly, and repeated if necessary. After blistering over the liver I content myself, in cases of pneumonia, with applying poultices over the lungs.

I hasten to publish these observations, for the reason that I understand this form of fever is largely prevalent in portions of Alabama and Georgia, with the hope that this method of treatment may be as useful in the hands of my professional brethren as it has been in mine, and thereby valuable lives may be saved.

